# Supplementary figures and images for: Biofilm-associated metabolism via ERG251 in Candida albicans
Source: PLoS Pathog. 2024 May 13;20(5):e1012225. doi: 10.1371/journal.ppat.1012225 (PMC11115363; doi:10.1371/journal.ppat.1012225)

Fig S1

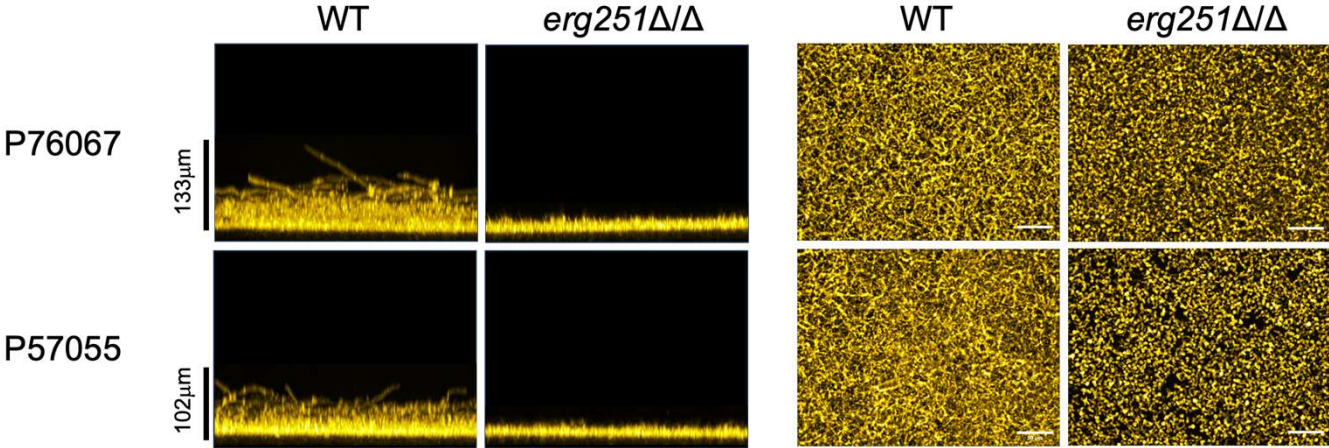

Supplement: S1 Fig — C. albicans wild type P57055 and P76067, and their respective erg251Δ/Δ mutants were assayed for biofilm formation under in vitro growth conditions. Strains were grown in RPMI+10% FBS in a 96-well plate at 37°C for 24 hours. Fixed biofilms were stained with calcofluor white and imaged using a Keyence BZ-X800E fluorescence microscope. Representative sections from each biofilm are shown. Scale bars indicate depth of the corresponding biofilm by wild type strain. White scale bars of apical view images indicate 50 μm in length. (PDF) [file ppat.1012225.s003.pdf]

Fig S2

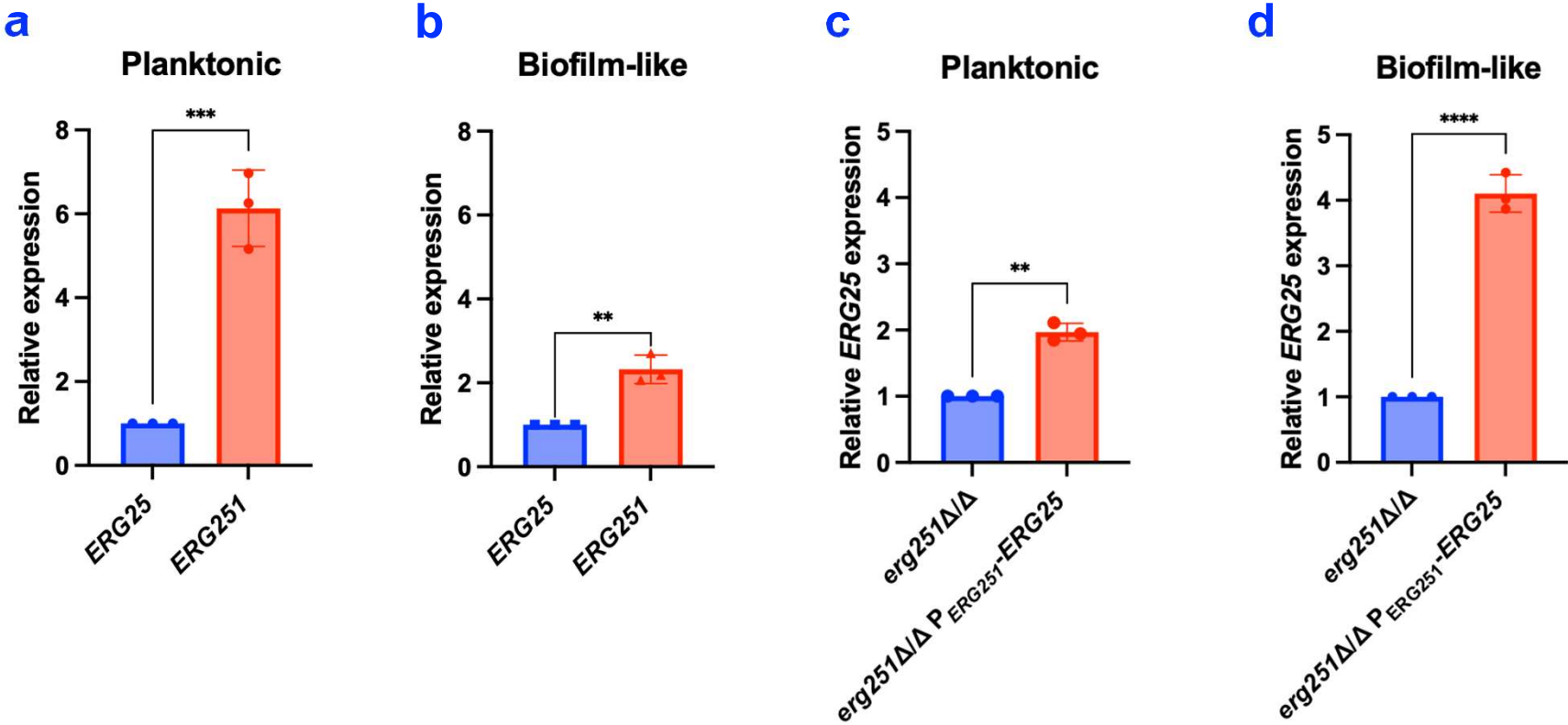

Supplement: S2 Fig — (a, b) Graph indicating relative mRNA level of ERG251 to ERG25. SC5314 wild type was grown in YPD at 30°C for 24 hours under planktonic (a) or biofilm-like (b) conditions. (c, d) Graph indicating relative ERG25 mRNA levels in ERG25 overexpression strain. erg251Δ/Δ mutant and erg251Δ/Δ PERG251-ERG25 were grown in YPD at 30°C for 24 hours under planktonic (c) and biofilm-like (d) conditions. RNAs of three independent biological samples were extracted for qPCR determination. Relative gene expression was compared using the threshold cycle ΔΔCT method. Statistical analysis was performed using T test. ** p-value < 0.01, *** p-value < 0.001, **** p-value < 0.0001. (PDF) [file ppat.1012225.s004.pdf]

Fig S3

**a**

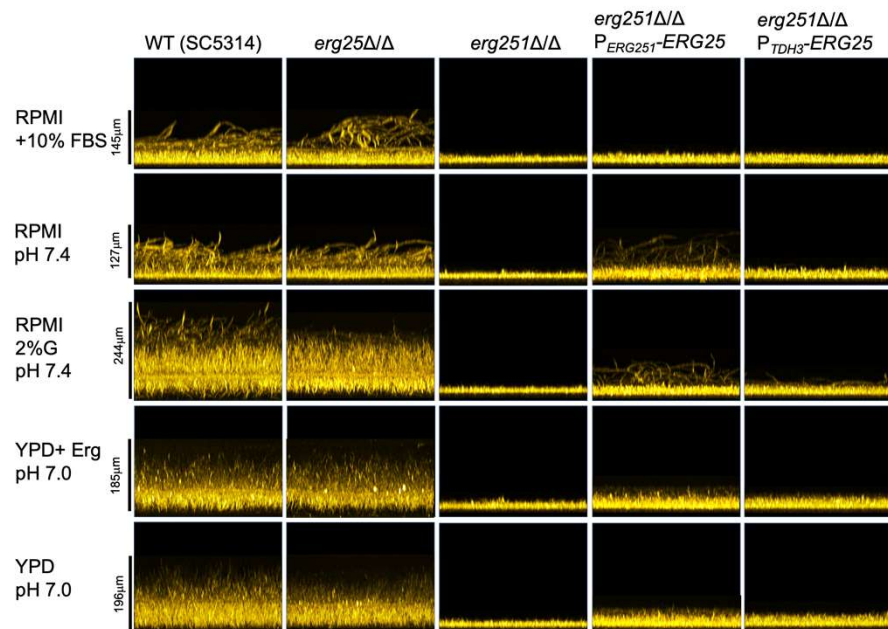

**b**

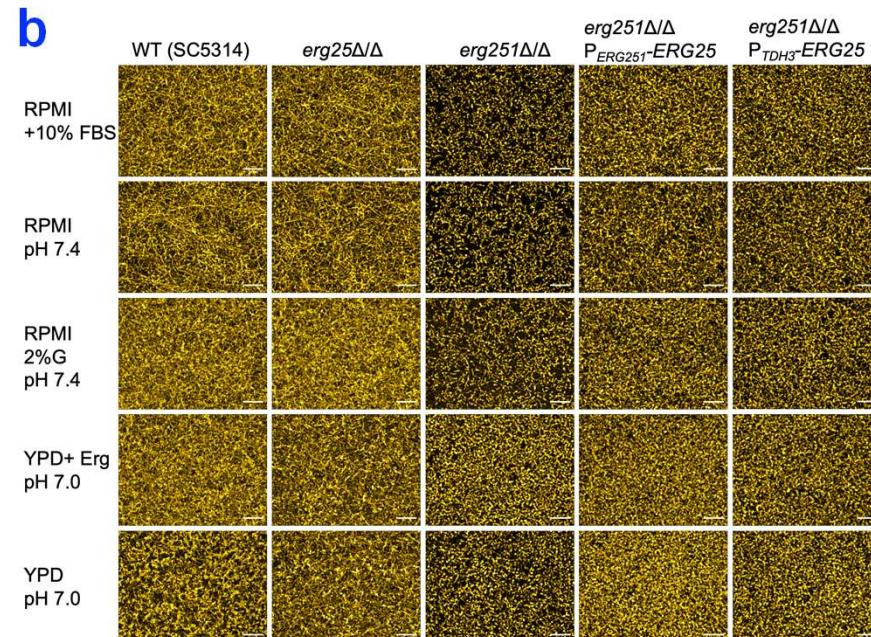

**c**

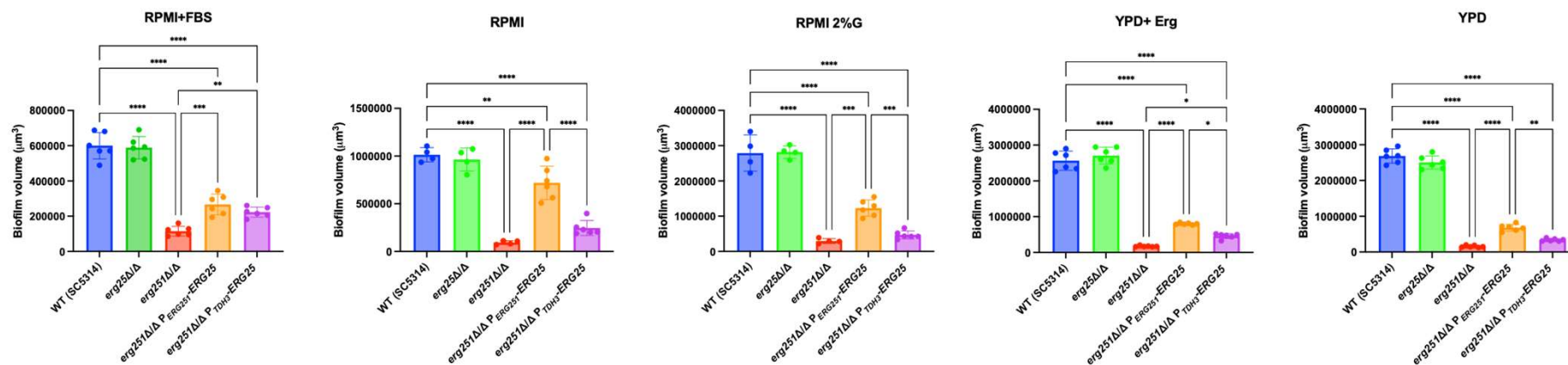

Supplement: S3 Fig — (a) Biofilm side-view projections. Five strains, including SC5314 wild type, erg25Δ/Δ, erg251Δ/Δ, PERG251-ERG25 erg251Δ/Δ, and PTDH3-ERG25 erg251Δ/Δ, were assayed for biofilm formation under in vitro growth conditions. Strains were grown in 5 media, including RPMI+10% FBS, RPMI 2% glucose (pH 7.4), RPMI (pH 7.4), YPD (pH 7.0) + ergosterol (0.002%), and YPD (pH 7.0), in a 96-well plate at 37°C for 24 hours. Fixed biofilms were stained with calcofluor white and imaged using a Keyence BZ-X800E fluorescence microscope. Scale bars indicate depth of the corresponding wild type biofilm. (b) Biofilm apical-view projections. Apical views of representative sections were generated with the same datasets used in (a). White scale bar indicates 50 μm in length. (c) Biofilm volume, measured with Image J and presented in column with 4 or 6 biologically independent samples. Statistical analysis was performed using one-way ANOVA. * p-value < 0.05, ** p-value < 0.01, *** p-value < 0.001, **** p-value < 0.0001. (PDF) [file ppat.1012225.s005.pdf]

Fig S4

a

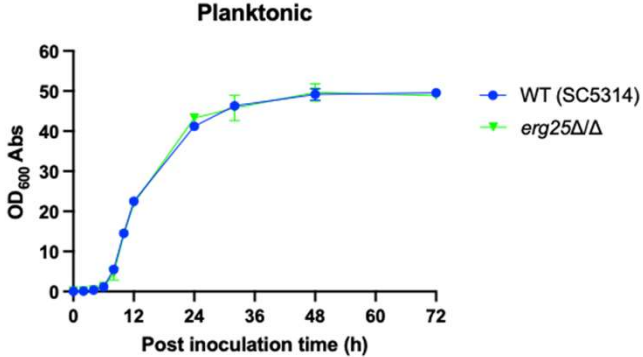

b

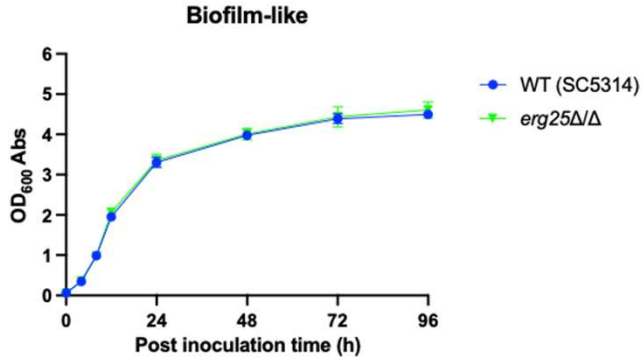

c

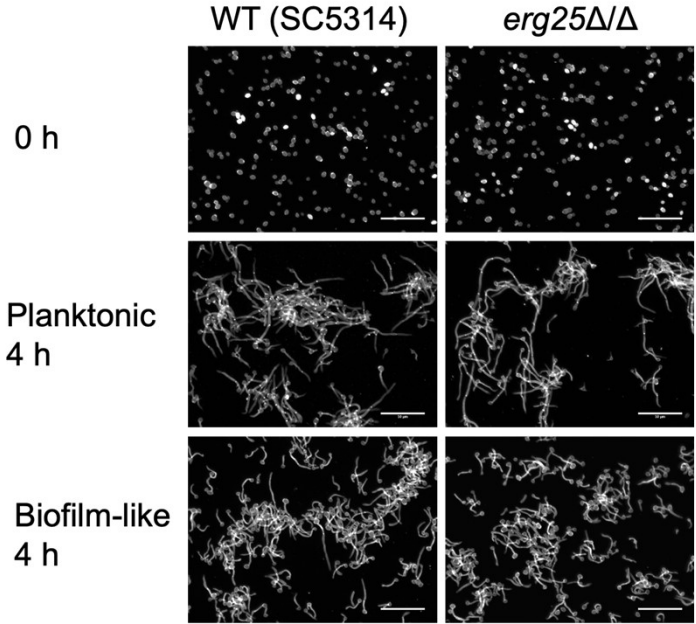

Supplement: S4 Fig — Growth phenotypes of SC5314 wildtype and erg25Δ/Δ were assayed in YPD medium at 30°C under planktonic (a) and biofilm-like (b) conditions, respectively. Mean values of OD600 Abs of triplicates at indicated time (hours post inoculation) were plotted using GraphPad Prism 10 software. And error bars represent the SEM. (c) Filamentation phenotypes were assayed in RPMI+10% FBS at 37°C for 4 hours under planktonic and biofilm-like conditions, respectively. Fixed ells were stained by calcofluor white and imaged by Zeiss fluorescence microscope. Representative images of each strain are shown. White scale bars indicate 50 μm in length. (PDF) [file ppat.1012225.s006.pdf]

Fig S5

**a**

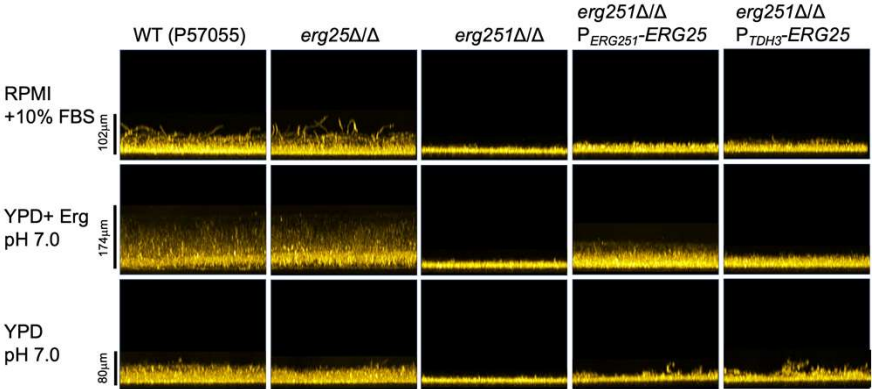

**b**

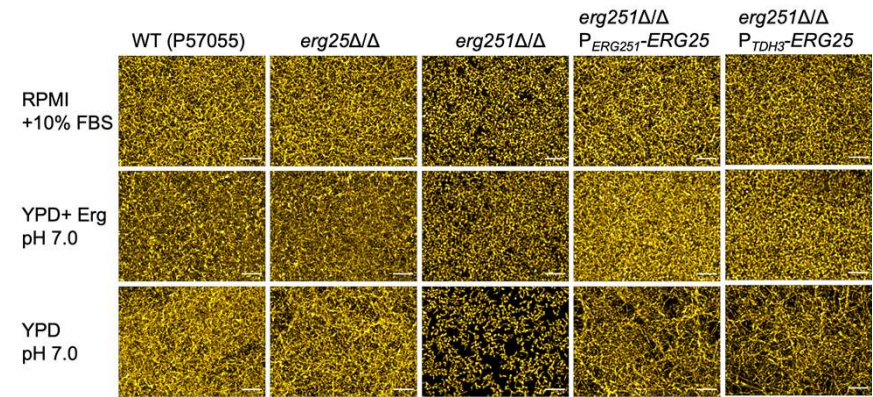

**c**

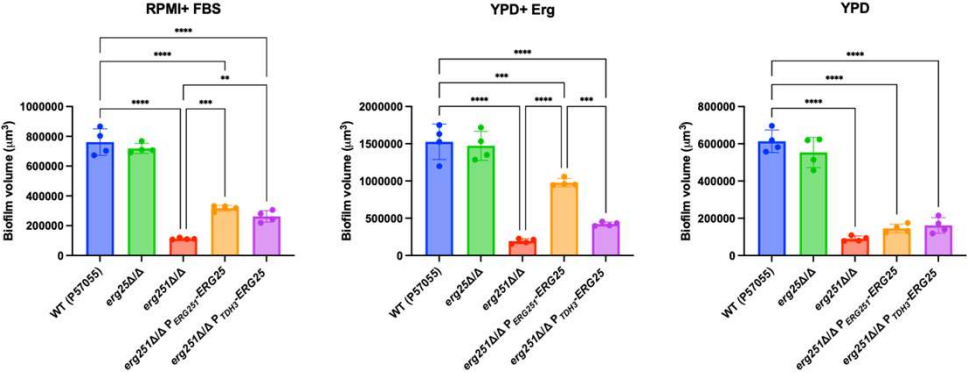

**d**

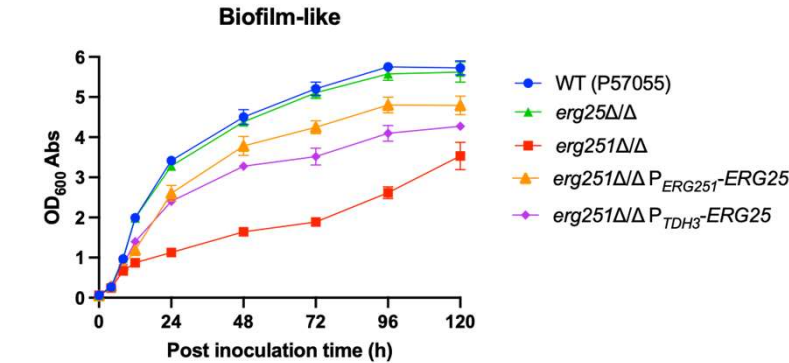

**e**

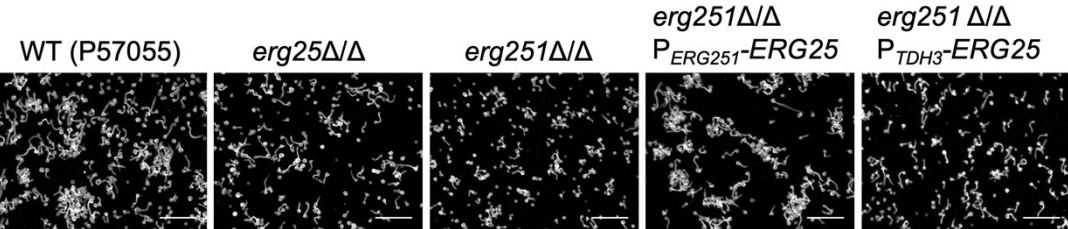

Supplement: S5 Fig — (a) Biofilm side-view projections. Five strains including P57055 wild type, erg25Δ/Δ, erg251Δ/Δ, erg251Δ/Δ PERG251-ERG25, and erg251Δ/Δ PTDH3-ERG25 were assayed for biofilm formation under in vitro growth conditions. Strains were grown in three media including RPMI +10% FBS, YPD (pH 7.0) + ergosterol (0.002%), and YPD (pH 7.0) in a 96-well plate at 37°C for 24 hours. Fixed biofilms were stained with calcofluor white and imaged using a Keyence BZ-X800E fluorescence microscope. Representative sections from each biofilm are shown. And relevant genotypes are given above each column. Scale bars indicate depth of the corresponding biofilm by wild type strain. (b) Biofilm apical-view projections. Apical views of representative sections were generated with the same datasets used in (a). White scale bars indicate 50 μm in length. (c) Biofilm volume, measured with Image J and presented in column with n = 4 biologically independent samples. Statistical analysis was performed using one-way ANOVA. ** p-value < 0.01, *** p-value < 0.001, **** p-value < 0.0001. (d) Growth phenotypes assayed under biofilm-like conditions. Each dot represents the average OD600 Abs of triplicates at indicated time. (e) Filamentation phenotypes assayed under biofilm-like conditions. Strains were grown in RPMI+10% FBS at 37°C for 4 hours. Fixed cells were stained by calcofluor white and imaged by Zeiss fluorescence microscope. Representative images of each strain are shown. White scale bars indicate 50 μm in length. (PDF) [file ppat.1012225.s007.pdf]

Fig S6

a

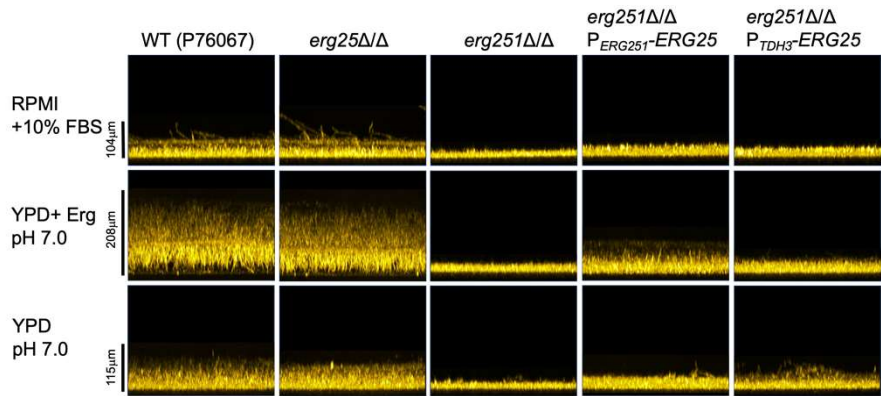

b

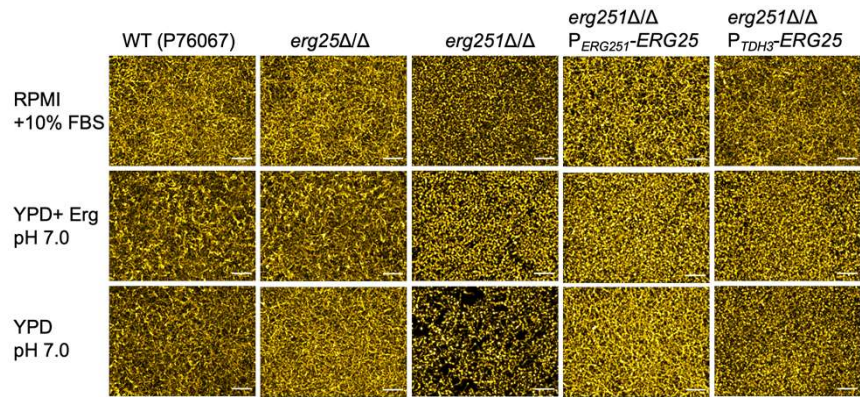

c

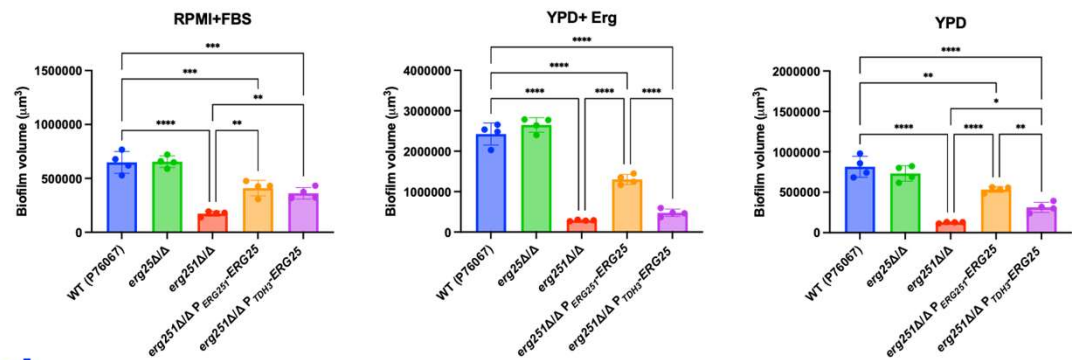

d

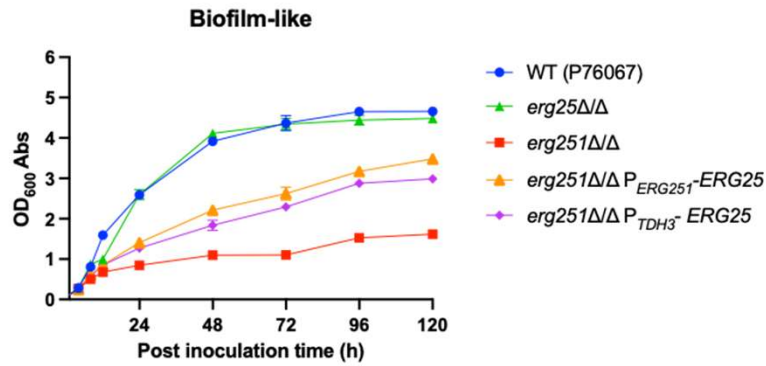

e

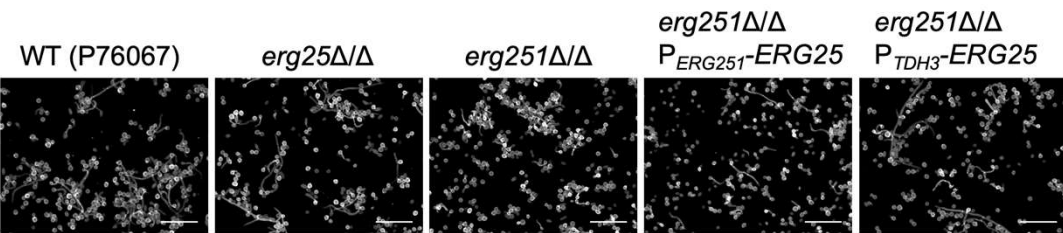

Supplement: S6 Fig — (a) Biofilm side-view projections. P76067 wild type, erg25Δ/Δ, erg251Δ/Δ, erg251Δ/Δ PERG251-ERG25, and erg251Δ/Δ PTDH3-ERG25 were assayed for biofilm formation under in vitro growth conditions. Strains were grown in three media, including RPMI +10% FBS, YPD (pH 7.0) with and without ergosterol (0.002%) in a 96-well plate at 37°C for 24 hours. Fixed biofilms were stained with calcofluor white and imaged using a Keyence BZ-X800E fluorescence microscope. Scale bars indicate depth of the corresponding biofilm by wild type strain. (b) Biofilm apical-view projections. Apical views of representative sections were generated with the same datasets used in (a). White scale bars indicate 50 μm in length. (c) Biofilm volume, measured with Image J and presented in column with n = 4 biologically independent samples. Statistical analysis was performed using one-way ANOVA. ** p-value < 0.01, *** p-value < 0.001, **** p-value < 0.0001. (d) Biofilm-like growth phenotypes. Each dot represents the average OD600 Abs of triplicates at indicated time. (e) Biofilm-like filamentation phenotypes. Strains were grown in RPMI +10% FBS at 37°C for 8 hours. Fixed ells were stained by calcofluor white and imaged by Zeiss fluorescence microscope. Representative images are shown. White scale bar represents 50 μm in length. (PDF) [file ppat.1012225.s008.pdf]
