# Supplementary material for: Biofilm-associated metabolism via ERG251 in Candida albicans
Source: PLoS Pathog. 2024 May 13;20(5):e1012225. doi: 10.1371/journal.ppat.1012225 (PMC11115363; doi:10.1371/journal.ppat.1012225)
Supplement: Attachment — Submitted filename: Response to reviewer comments.docx [file ppat.1012225.s009.docx]

***********************

Reviewer Comments (if any, and for reference):

Reviewer's Responses to Questions

Part I - Summary

Please use this section to discuss strengths/weaknesses of study, novelty/significance, general execution and scholarship.

Reviewer #1: In this paper, Xiong and colleagues investigate the role of ERG251, due to its conserved regulatory relationship with the Efg1 transcription factor in multiple strains of C. albicans. As Efg1 controls biofilm formation in multiple strains, the authors demonstrate that Erg251 is a critical part of that regulation. The phenotype of the erg251 strain is only revealed under biofilm conditions, with accumulation of alternate sterols in the membrane under hypoxia. The authors also demonstrate that Erg25, a duplicated gene, can partially compensate for loss of Erg251 in biofilm formation, but only when it is overexpressed.

This is a clear and well written paper. My main concern is about the model in figure 7, which I think needs additional data.

Thank you for this very positive summary. I address the specifics of your concerns below in response to your detailed comments. I hope you agree that we have addressed the concerns adequately.

Reviewer #2: This manuscript presents a very thorough analysis of the role of the Erg251 gene in controlling biofilm-specific ergosterol regulation during biofilm formation and shows that this gene is necessary for biofilm formation, but not for planktonic growth. The authors study this gene's role in multiple C. albicans strains and find similar results throughout. They demonstrate that loss of Erg251 leads to biofilm-specific loss of ergosterol and accumulation of intermediates. They also demonstrate that the Erg25 parlog does not play a role in this phenotype, but if over expressed it can partially complement loss of Erg251. The one minor weakness that I noted was they measured Erg25 overexpression in Figure S2c, but only in planktonic conditions. Biofilm conditions would be more relevant given the emphasis of this relationship in the manuscript,

Thank you for this clear and highly supportive summary. We have included the RNA measurements you mention in a revised figure S2 with addition of a new panel d.

Reviewer #3: This paper approaches the question of why Candida albicans and related yeasts have two genes for C-4 sterol methyl oxidases (Erg25 and Erg251) while other fungi and algae can get by with only one. The authors do a thorough and convincing job of showing that ERG251 is required for growth under partially anaerobic conditions (biofilms) but not under fully aerobic conditions (planktonic cells). This observation expands on Candida's ability to thrive under many different growth conditions requiring metabolic flexibility. These growth studies are also integrated with changes in fluconazole sensitivity, which are interpreted appropriately in terms of changing levels of carbon flow through slightly altered paths for sterol biosynthesis. That's all to the positive.

I am very grateful for this excellent and positive summary.

The following are items that might be addressed for improved clarity and impact.

1/ The paper mentions planktonic versus biofilm conditions repeatedly, and the Discussions focused on this distinction almost exclusively. Yet your standard conditions are really only comparing cells grown in YPD with shaking versus YPD sealed without shaking, with the latter being anaerobic to varying degrees as growth proceeds. I have no problem with the practicality of this distinction, and you cite references for the strong overlap of genes turned on for hypoxia and biofilms. It would be nice if you made an effort to distinguish hypoxia/anaerobic genes from biofilm genes. Would your mutants defective in erg25 or erg251 grow in liquid anaerobic conditions, such as those described by Dumitru et al AAC 2004?

Thank you for this suggestion. During the course of our studies, Leah Cowen mentioned that her group is conducting [her words, with her permission] “a very focused study on ERG251 in anaerobic conditions for which we are on the home stretch.” She believes that “our paper will be highly complementary to yours.” Hence let me ask your forbearance here so that we avoid investing time to duplicate what is certain to be a high-quality study from the Cowen group on the topic of anaerobic growth and Erg251/Erg25.

2/ The theme that ERG251 provides C. albicans with greater flexibility under anaerobic conditions (gut) could easily be tested if you switched from YPD to a chemically defined growth medium (RPMI??).

Apologies; we may have been too subtle in our descriptions. We did use two additional media in our study:

(a) For our filamentation assays, we used RPMI+FBS. Those experiments are in Figure 2, panels e and f. The *erg251* mutant was severely defective in filamentation in RPMI+FBS under those conditions, as would be expected from a growth defect. Filamentation was restored in RPMI+FBS when *ERG25* was overexpressed (Figure 4, panels c and d). And the *erg25* mutant had no filamentation defect (Figure S4, panel c). I admit that the addition of FBS means that the medium is not really chemically defined, but it is necessary to induce filamentation in many of the non-SC5314 isolates with which we validated our results. In any case, at least it is not YPD!

(b) For our GC-MS samples, we grew the cells in defined CSM medium for 24 hr, 30°C, as indicated in the Figure 5 legend. The cells grew as yeast. OD600 measurements indicate that growth yield of the WT was reduced about 3-fold under biofilm like conditions compared to planktonic conditions, and that the *erg251* mutants remained severely growth-defective under biofilm-like conditions:

I hope that you agree then that our findings regarding the growth defect of *erg251*Δ/Δ mutants are not restricted to YPD.

Andreasen and Stier (J Cell Physiol 1953) showed that S. cerevisiae had a nutritional requirement for ergosterol anaerobically. Please add this reference to line 303.

Done! Thank you.

When looking at similar conditions for C. albicans, Dumitru et al expected that Candida too would require ergosterol, but it did not. They were unable to provide a good explanation for how C. albicans differed in this regard. Now you could suggest that having both ERG251 and ERG25 provides the answer, and you have the mutants to prove it.

The Dumitru study is fascinating. However, developing this theme in our study will extend the scope well beyond our focus on biofilm formation, and will cause us to duplicate work from the Cowen lab. As you may know, I have provided strains to the entire Candida community, generally within 2 weeks of any request, at a clip of about 20-50 different labs per year for the last 25 years. If your lab wanted to test your own hypothesis with our strains, all you’d have to do is send a strain request by email.

3/ I like your experiments Figure 5a and S7 exploring the sterols produced under your "biofilm" conditions, but it troubled me to see them left as Sterol A and sterol B and later referred to as "novel" instead of not yet identified.

Apologies – I agree that “novel” is not accurate. I found two places where we used that term, lines 297 and 1018 in the initial submission. I have changed “novel” in both cases to "unidentified."

They do not match eburicol or 4,4 dimethyl zymosterol. But Erg 25 and Erg251 are 4-methyl oxidases, and in other organisms there are other potential substrates that could accumulate in the erg251 double mutant. Ergosterol is also the dominant sterol in Chlamydomonas reinhardtii (Brumfield, Eur J Phycol 2017) and Chloella variabilis (Voshall, J Phycol 2021). These pathways to ergosterol differ from the yeasts in some significant ways but significantly, in one putative sterol pathway, four sterols are alternate substrates for a single C-4 methyl oxidase, three of which seem possible for C. albicans grown under hypoxic conditions: obtusifoliol, 24-dihydroobtusifoliol, and 24 methylene lophenol. Please forgive me if you have already made extensive efforts to identify these sterols and they are in fact novel. I would love to see Fig S7 incorporated into Fig 5a.

Thank you for those excellent ideas! We have taken your suggestion and include the graphs from the previous Figure S7 in an expanded Figure 5 and modified the figure legend accordingly.

Part II – Major Issues: Key Experiments Required for Acceptance

Please use this section to detail the key new experiments or modifications of existing experiments that should be absolutely required to validate study conclusions.

Generally, there should be no more than 3 such required experiments or major modifications for a "Major Revision" recommendation. If more than 3 experiments are necessary to validate the study conclusions, then you are encouraged to recommend "Reject".

Reviewer #1: The model in figure 7 suggests that both Erg25 and Erg251 are well expressed under planktonic conditions and that there is compensation that cannot be achieved in biofilm conditions when Erg251 is lost. In Figure S2, it looks like Erg251 is the major paralog in both conditions, as its expression is higher than Erg25 in both. If anything, the relative abundance is much higher in planktonic, so the phenotype of the mutant should be more apparent in planktonic conditions.

The prediction that an *erg251* mutant should have a stronger defect during planktonic growth rests on the assumption that the methyl oxidase step is rate-limiting under planktonic conditions. That assumption is probably not true though. In the 2020 review *Regulation of Ergosterol Biosynthesis in Saccharomyces cerevisiae* by Jordá and Puig it is indicated that, under conventional growth conditions, the rate-limiting steps are HMG-CoA reductase, Squalene epoxidase, and Lanosterol 14-alpha-demethylase (Erg11). The review has been cited over 100 times so I take it to represent mainstream thought.

Figure legend 7 says “higher expression levels of ERG251 relative to ERG25 may contribute to differences in function of the two paralogs under biofilm conditions” is not well supported by the current data.

We have expanded the Figure 7 legend to accommodate several possible scenarios, as follows, starting at line 921 in the initial submission. (I have included the entire legend for context and underlined the major change.)

**“****Fig 7. Functions of *ERG251* and *ERG25* in ergosterol metabolism.** *C. albicans* has two paralogs that specify C-4 sterol methyl oxidase, *ERG251* and *ERG25*. Under planktonic conditions, either Erg251 or Erg25 is sufficient to support ergosterol synthesis. Under biofilm conditions, Erg251 is required for ergosterol synthesis at levels adequate for growth and filamentation. Biofilm conditions may impair ergosterol synthesis through limitation of oxygen and iron. *ERG251* may be more critical than *ERG25* for growth under biofilm conditions due to differences in expression levels, substrate affinities, catalytic rates, or ability to assemble into the C-4 demethylation complex [41, 63] with Erg26, Erg27, and Erg28.

The argument that biofilm conditions limit ergosterol biosynthesis should be further supported by comparing ergosterol levels in the WT between conditions,

Excellent point! The ergosterol levels are similar in the wild-type strain under the two conditions, as seen by comparison of Figure 5, panels b and c in the initial submission. Now let us take that idea one step further and calculate the rate of ergosterol accumulation in the two situations, which is more on point for your criticism. Our estimate is that the rate of accumulation of ergosterol is about 5 times slower in biofilm-like conditions than in planktonic conditions.

Planktonic conditions:

We measured 74.17 pmol ergosterol per 5*10exp8 cells.

Half of the cells were created in the last division before harvest, so it stands to reason that half of the ergosterol accumulated during the last division as well. In that case we accumulated 37 pmol per 2.5*10exp8 cells.

That last division took 1.5 hrs, the approximate doubling time in planktonic conditions.

So the rate of accumulation of ergosterol in our WT cells in planktonic conditions is

37 pmol per 2.5*10exp8 cells/1.5 hr = 10 pmol per hr per 10exp8 cells

Biofilm-like conditions:

We measured 83.57 pmol ergosterol per 5*10exp8 cells.

Following the same logic, we accumulated 42 pmol per 2.5*10exp8 in the last division.

That last division took 8 hr, the approximate doubling time in biofilm-like conditions.

So the rate of accumulation of ergosterol in our WT cells in biofilm-like conditions is

42 pmol per 2.5*10exp8 cells/8 hr = 2.1 pmol per hr per 10exp8 cells.

and by analyzing ERG25 and ERG251 transcripts in the WT between conditions.

The transcript levels are analyzed in the wild type and the ERG25 overexpressors in Figure S2. The results show that *ERG251* RNA levels are higher than *ERG25* RNA levels under both conditions, and confirm published results from Lu et al. mBio. 2023 Feb 28;14(1):e0263922.

Additionally, the authors should make and characterize the Erg25 Erg251 double mutant strain.

We have attempted to make the double mutant repeatedly. It is expected to be Erg- so we included ergosterol and Tween in the transformation plates. To improve transformation rates we used CRISPR methods, and to hedge our bets we started independently with *erg25* and *erg251* single mutants. We have been unable to obtain the double mutant. We can get *erg251Δ/Δ erg25Δ/+* and *erg251Δ/+ erg25Δ/Δ* transformants, so the DNA is getting in there. We suspect that ergosterol uptake is not adequate on our selective media. Thus we are unable to make the double mutant, despite several attempts.

This result was to be expected, based on Martin Bard’s published studies of *ERG26* and *ERG27*, which encode other methylase complex components. His lab was unable to recover homozygous mutants of either gene, despite including ergosterol in the medium (PMID: 12702354; PMID: 15552648).

The authors should also perform a standard CLSI methods test for fluconazole susceptibility of the different mutant strains. As C. albicans grows well in 96-well plates, this would allow for a clearer and more quantitative measure of fluconazole susceptibility.

In the original report of *efg1*Δ/Δ mutant susceptibility to azoles, *Morphogenic regulator EFG1 affects the drug susceptibilities of pathogenic Candida albicans*, Prasad and colleagues remarked, “Notably, the observed enhanced susceptibility of the Δefg1 mutant was only confined to cells when grown on a solid agar surface and not when grown in liquid media.” Elsewhere in the paper they mentioned that they used the NCCLS method, and saw no difference. We nonetheless tried a CLSI test with our own strains. The results, shown below, are in keeping with Prasad’s earlier studies.

Reviewer #2: There are no major issues.

Reviewer #3: None

Part III – Minor Issues: Editorial and Data Presentation Modifications

Please use this section for editorial suggestions as well as relatively minor modifications of existing data that would enhance clarity.

Reviewer #1: The alternate sterols produced by the mutants are quite interesting, and I would recommend moving SFig7 a into the main figure 5.

Done!

Reviewer #2: I think they should measure ERG25 expression in the epistasis strains in biofilm conditions.

Done!

Reviewer #3: My feeling is that the paper is longer than needed for the points being made, but since I have no concrete suggestions on what should be cut, I haven't said so in my comments to the authors. I mention it only in case the other reviewer(s) do make such suggestions.

In view of this comment I have gone through the manuscript with an eye toward condensing and deleting words and sentences. I managed to cut around 3-4 sentences. We also have eliminated Figure S7 by moving it into Figure 5. We are close to the minimum as I see it, but I am open minded to any further suggestions. There is nothing worse than reading a paper that is longer than necessary!
